# Nicotine suppresses apoptosis by regulating α7nAChR/Prx1 axis in oral precancerous lesions

**DOI:** 10.18632/oncotarget.20506

**Published:** 2017-08-24

**Authors:** Chunxiao Wang, Wenwen Niu, Hui Chen, Ni Shi, Dian He, Min Zhang, Lihua Ge, Zhenchuan Tian, Moci Qi, Tong Chen, Xiaofei Tang

**Affiliations:** ^1^ Division of Oral Pathology, Beijing Institute of Dental Research, Beijing Key Laboratory, Beijing Stomatological Hospital & School of Stomatology, Capital Medical University, Beijing, China; ^2^ Division of Medical Oncology, Department of Internal Medicine, The Arthur G. James Cancer Hospital and Richard J. Solove Research Institute, The Ohio State University, Columbus, OH, USA; ^3^ Department of Epidemiology and Health Statistics, School of Public Health, Capital Medical University, Beijing, China

**Keywords:** oral precancerous lesion, Prx1, nicotine, α3nAChR, α7nAChR

## Abstract

Nicotine, a tumor promoter in tobacco, can increase Peroxiredoxin (Prx1) and nicotinic acetylcholine receptors (nAChRs) in oral squamous cell carcinoma (OSCC). In the present study, we investigate the effects of nicotine in oral precancerous lesions focusing on apoptosis and nAChR/Prx1 signaling. We detected expression of Prx1, α3nAChR, α7nAChR, phosphorylation of mitogen-activated protein kinases (MAPK) and apoptosis in dysplastic oral keratinocyte (DOK) cells as well as in 4-nitroquinoline 1-oxide (4NQO) or 4NQO + nicotine – induced oral precancerous lesions in Prx1 wild-type (Prx1^+/+^) and Prx1 knockdown (Prx1^+/-^) mice. In DOK cells, Prx1 knockdown and blocking α7nAChR activated apoptosis, and nicotine increased the expression of Prx1, α3nAChR and α7nAChR, and inhibited MAPK activation. Moreover, nicotine suppressed apoptosis depending on Prx1 and α7nAChR in DOK cells. In animal bioassay, nicotine and Prx1 promoted growth of 4NQO-induced precancerous lesions in mouse tongue. 4NQO plus nicotine suppressed MAPK activation in Prx1 wild-type mice but not in Prx1 knockdown mice. Our data demonstrate that nicotine inhibits cell apoptosis and promotes the growth of oral precancerous lesions via regulating α7nAChR/Prx1 during carcinogenesis of OSCC.

## INTRODUCTION

Oral cancer is the sixth most common malignant tumors worldwide, with more than 90% of oral cancers in squamous cell carcinoma type [1]. There are approximate 500,000 new cases diagnosed with oral squamous cell carcinoma (OSCC) annually and more than 250,000 cases died every year [2]. The overall 5-year survival rate of oral cancer remains around 50% despite the advances of radiotherapy and chemotherapy in recent decades [3]. Oral leukoplakia (OLK) is one of the most prevalent oral precancerous lesions of OSCC and increases the risk of OSCC development at a malignant transformation rate of 0.13-17.5% [4, 5]. Therefore, it is critical to monitor OLK development and reduce the prevalence of OSCC through reversal of OLK by clinical interventions.

Etiologically, tobacco is one of the most important risk factors in OLK pathogenesis [5]. Nicotine, a tumor promoter in tobacco, contributes to tumorigenesis mainly by promoting growth and survival of mutated cancer cells and protecting them from apoptosis by creating a tumor favorable environment [6]. *In-vitro* studies showed that nicotine inhibits apoptosis in oral cancer [7, 8]. Nicotinic acetylcholine receptors (nAChRs) play an important role in nicotine induced tumorigenesis [9]. nAChRs ligands gate across the plasma membrane ion channel receptors and are composed of various subunits of homologous or heterologous polymers [9]. nAChR subtypes α2, α3, α4, α5, α7 and α9 are identified in oral epithelial cells [10]. The activation of α3, α4, α7 and α9nAChR can produce a combination effect of growth-promoting and anti-apoptotic signals [11]. α7nAChR is the main subtype receptor of tobacco products [12]. The alteration of α7nAChR accompanied by induced epidermal growth factor (EGF), phosphatidylinositol-4,5-bisphosphate 3-kinase (PI3K), cyclin D1, extracellular signal-regulated kinase (ERK1/2) and inhibition of mitochondrial permeability transition pore (mPTP) opening facilitates tumor promotion and progression [13-15]. Studies show that long-term use of nicotine enhances cancer cell migration and invasion with morphological alterations, and inhibition of α7nAChR may provide a feasible approach for preventing the progression of head and neck cancer [16]. α3nAChR is another key acetylcholine receptor in oral epithelial cells. Studies suggest that α3nAChR gene silencing and α3nAChR antagonist inhibit nicotine-induced cell proliferation in oral gingival epithelial cells [17].

In our previous studies, we observed an overexpression of Peroxiredoxin 1 (Prx1), α3 and α7nAChRs in SCC15 cells exposed to nicotine [18]. We also found that Prx1 was involved in OLK pathogenesis by providing resistance against extracellular damages from oxidative stress via inhibition of apoptosis signal-regulating kinase 1 (ASK1) [19]. However, the data about the functional role of Prx1 in nicotine-related oral precancerous lesions are limited. In the current study, we assessed nAChR/Prx1 axis *in vitro* and in 4-nitroquinoline 1-oxide (4NQO) or 4NQO + Nicotine – induced oral precancerous lesions in wild-type (Prx1^+/+^) and Prx1 knockdown (Prx1^+/-^) mice.

## RESULTS

### Nicotine upregulates the expression of Prx1, α3nAChR and α7nAChR *in vitro*

**Figure 1:**
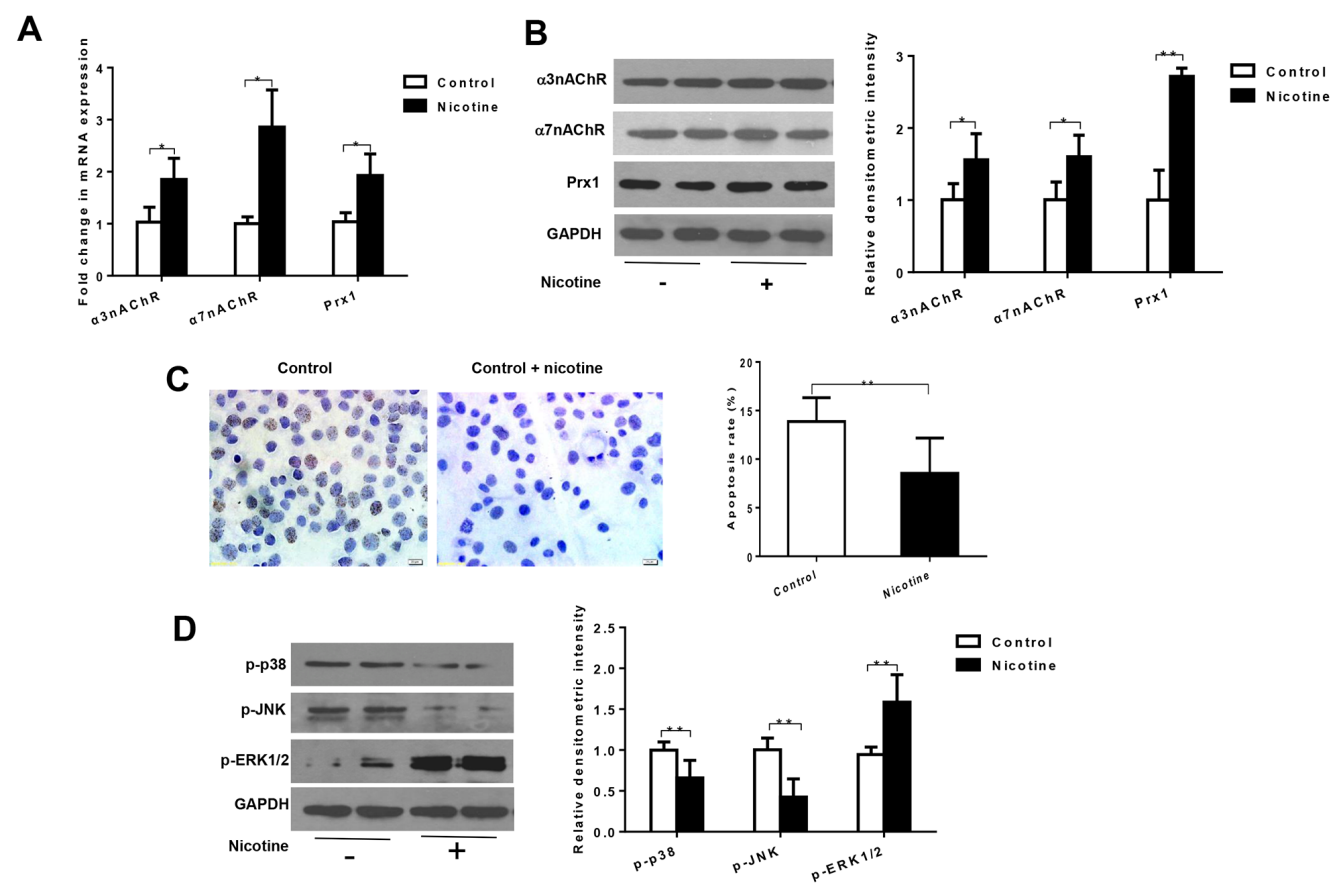
Nicotine increases expression of α3nAChR, α7nAChR and Prx1, and inhibits apoptosis in DOK cells **(A)** mRNA expression of α3nAChR, α7nAChR and Prx1; **(B)** protein expression of α3nAChR, α7nAChR and Prx1; **(C)** apoptosis rate detected by TUNEL; and **(D)** phosphorylation of p38, JNK and ERK1/2. The values are expressed as mean; *bars*, ± SE. * *P* < 0.05; ** *P* < 0.01.

In oral precancerous cell line DOK cells, nicotine significantly increased the mRNA expression of Prx1, α3nAChR and α7nAChR when compared to control cells (*P* < 0.05, Figure 1A). Similar results were observed in protein expression of Prx1 (*P* < 0.01), α3nAChR and α7nAChR (*P* < 0.05, Figure 1B).

### Nicotine suppresses apoptosis and modulates phosphorylation of p38, JNK and ERK *in vitro*

As shown in Figure 1C, nicotine significantly inhibited apoptosis in DOK cells (*P* < 0.01; Figure 1C). Nicotine also decreased the expression of p-p38 and p-JNK and increased expression of p-ERK1/2 in DOK cells (*P* < 0.01; Figure 1D).

### Nicotine suppresses apoptosis depending on Prx1 *in vitro*

**Figure 2:**
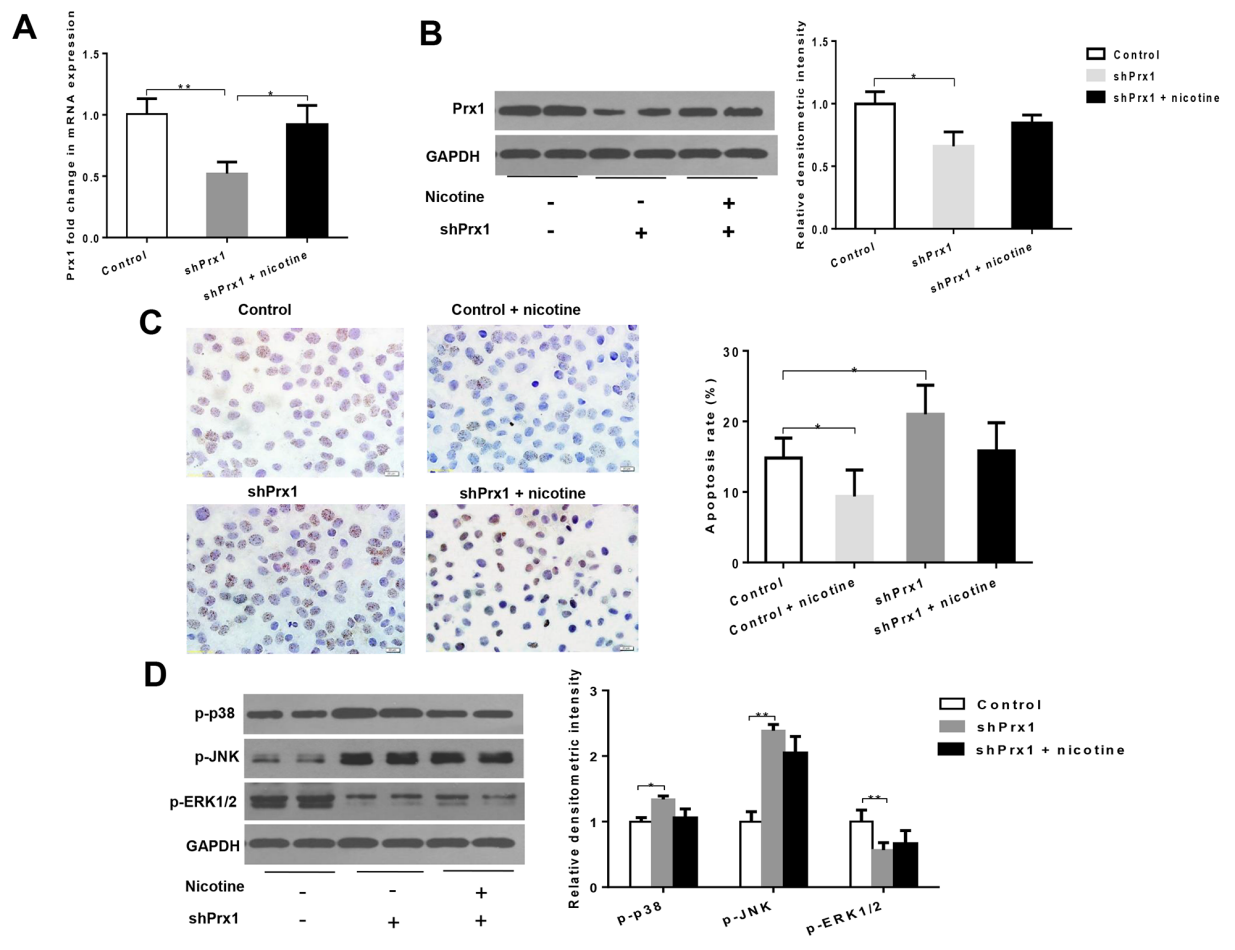
Effects of Prx1 knockdown on Prx1, apoptosis and MAPK in DOK cells **(A)** mRNA expression of Prx1; **(B)** protein expression of Prx1; **(C)** apoptosis rate detected by TUNEL; and **(D)** phosphorylation of p38, JNK and ERK1/2. The values are expressed as mean; *bars*, ± SE. * *P* < 0.05; ** *P* < 0.01.

As shown in Figure 2A and 2B, both mRNA and protein expression levels of Prx1 were significantly decreased by Prx1 Knockdown in DOK cells. Prx1 knockdown significantly increased cell apoptosis (*P* < 0.05) and expression of p-p38 and p-JNK (*P* < 0.05), and decreased expression of p-ERK1/2 when compared to control cells (*P* < 0.01; Figure 2C and 2D). Nicotine significantly inhibited apoptosis in control cells (*P* < 0.05) but not in Prx1 knocked down cells (Figure 2C). Similarly, nicotine did not exhibit significant effects on phosphorylation of p38, JNK and ERK1/2 in Prx1 knocked down cells (Figure 2D).

### Nicotine suppresses apoptosis depending on α7nAChR but not α3nAChR *in vitro*

**Figure 3:**
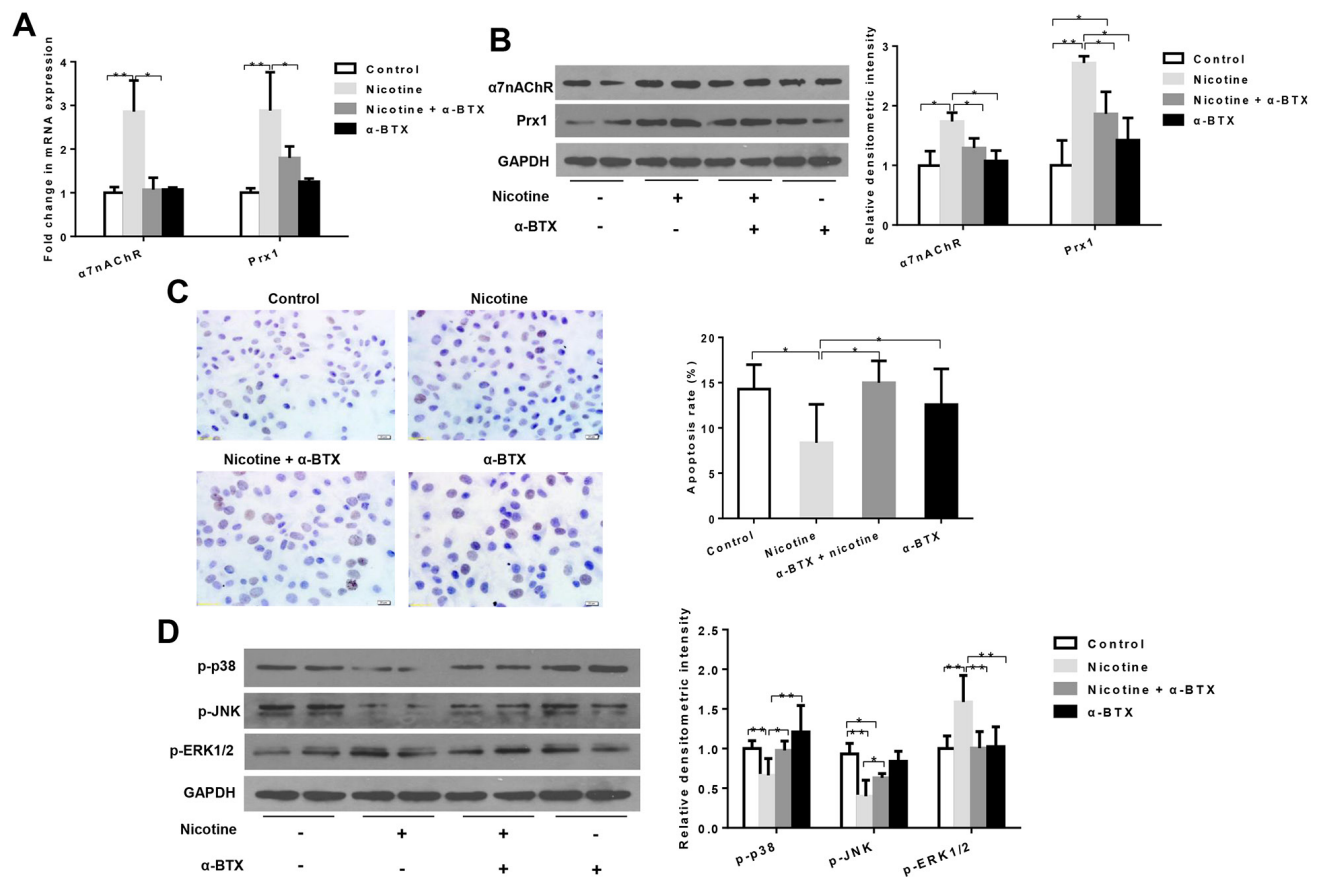
Effects of specific inhibition of α7nAChR on Prx1, apoptosis and MAPK in DOK cells **(A)** mRNA expression of α7nAChR and Prx1; **(B)** protein expression of α7nAChR and Prx1; **(C)** apoptosis rate detected by TUNEL; and **(D)** phosphorylation of p38, JNK and ERK1/2. The values are expressed as mean; *bars*, ± SE. * *P* < 0.05; ** *P* < 0.01.

The combination treatment of nicotine and α-BTX (a specific inhibitor of α7-nAChR) significantly reduced mRNA and protein expression of α7nAChR when compared to cells treated with nicotine only (*P* < 0.05; Figure 3A and 3B). In addition, the expression level of Prx1 was also decreased, which was similar to that observed with α7nAChR (*P* < 0.05; Figure 3A and 3B). As shown in Figure 3C, the apoptosis rate was increased in cells treated with nicotine + α-BTX when compared to those treated with nicotine only (*P* < 0.05). The expression of p-p38 and p-JNK was increased and p-ERK1/2 was decreased in cells treated with nicotine + α-BTX when compared to those treated with nicotine only (Figure 3D). Knockdown α3nAChR in DOK cells did not exhibit any effects on Prx1 and apoptosis (Supplementary Figure 1).

### 4NQO induces tongue precancerous lesions in mice

**Figure 4:**
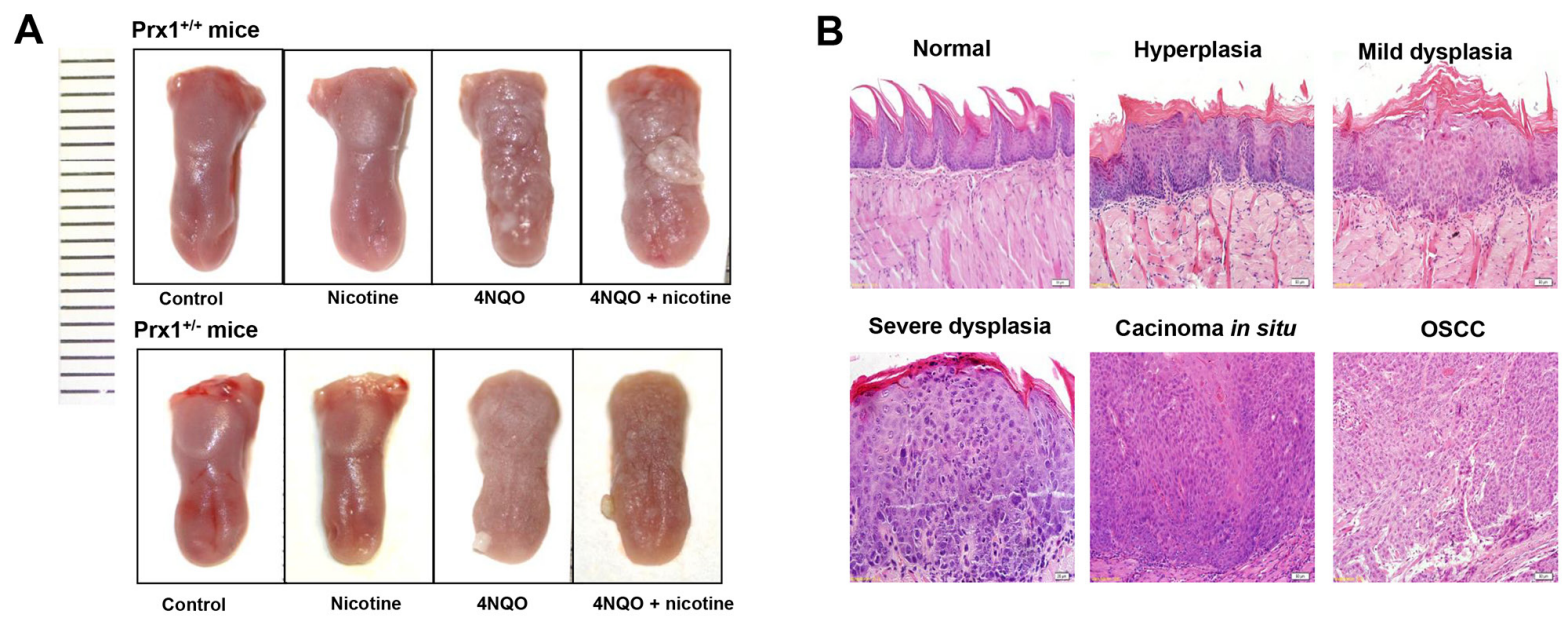
Histopathology of mouse tongue in animals treated with nicotine, 4NQO or ANQO plus nicotine **(A)** the representative images of entire tongue collected from Prx1 wild-type and Prx1 knockdown mice; and **(B)** the representative H&E staining of mouse tongue including normal, hyperplasia, dysplasia, carcinoma *in situ* and OSCC (magnification x 200).

In 4NQO or 4NQO + nicotine –treated animals, the tongues exhibited generally white, thick, rough and visible white patches as well as surface toughness (Figure 4A). Histologically, 4NQO or 4NQO + nicotine-induced epithelial hyperplasia and dysplasia had a thickening keratin layer, similar to OLK histologic features (Figure 4B). We also observed carcinomas *in situ* and OSCC in animals treated with 4NQO or 4NQO + nicotine (Figure 4B).

### Nicotine and Prx1 promote the progression of tongue precancerous lesions

As shown in Table 1, mice treated with nicotine alone (Groups 3 and 4) developed epithelial hyperplasia but no dysplasia or tumors. 4NQO treatment (Groups 5 and 6) induced hyperplasia, dysplasia (mild, moderate and severe) and tumors in mouse tongue mucosa. In wild-type mice, the tumor incidence increased from 5% in animals treated with 4NQO only (Group 5) to 40% in those treated with 4NQO + nicotine (Group 7; *P* < 0.05). In animals treated with 4NQO + nicotine, the incidences of severe dysplasia and tumors were significantly lower in Prx1 knockdown mice (Group 8) compared to wild-type mice (Group 7; *P* < 0.05).

**Table 1:** Histological evaluation of mouse tongue in animals treated with nicotine, 4NQO or 4NQO plus nicotine

Group	Treatment	No. of animals	Normal	Hyperplasia	Dysplasia			Carcinoma *in situ/*OSCC
					Mild	Moderate	Severe	
1	Prx1^+/+^^*a*^	10	10 (100%)					
2	Prx1^+/-*b*^	10	10 (100%)					
3	Prx1^+/+^ + nicotine^*c*^	20	15 (75%)	5 (25%)				
4	Prx1^+/-^ + nicotine	20	18 (90%)	2 (10%)				
5	Prx1^+/+^ + 4NQO^*d*^	20		2 (10%)	11 (55%)	2 (10%)	4 (20%)	1 (5%)
6	Prx1^+/-^ + 4NQO	20		4 (20%)	9 (45%)	6 (30%)	1 (5%)	
7	Prx1^+/+^ + 4NQO + nicotine^*e*^	20		1 (5%)	6 (30%)	3 (15%)	5 (25%)	5 (40%)^*f*^
8	Prx1^+/-^ + 4NQO + nicotine	20		3 (15%)	8 (40%)	6 (30%)	2 (10%)^*g*^	1 (5%)^*g*^

^*a*^ Wild-type C57BL/6 mouse.

^*b*^ Prx1 knockout mouse.

^*c*^ 5% nicotine smeared on tongue mucosa, 3 times per week.

^*d*^ 50 μg/mL 4NQO smeared on tongue mucosa every day.

^*e*^ Nicotine plus 4NQO treatment.

^*f*^***P*** < 0.05 compared to Group 5.

^*g*^***P*** < 0.05 compared to Group 7.

### Nicotine plus 4NQO increases the expression of Prx1, α3nAChR and α7nAChR, and induces apoptosis in wild-type mice

**Figure 5:**
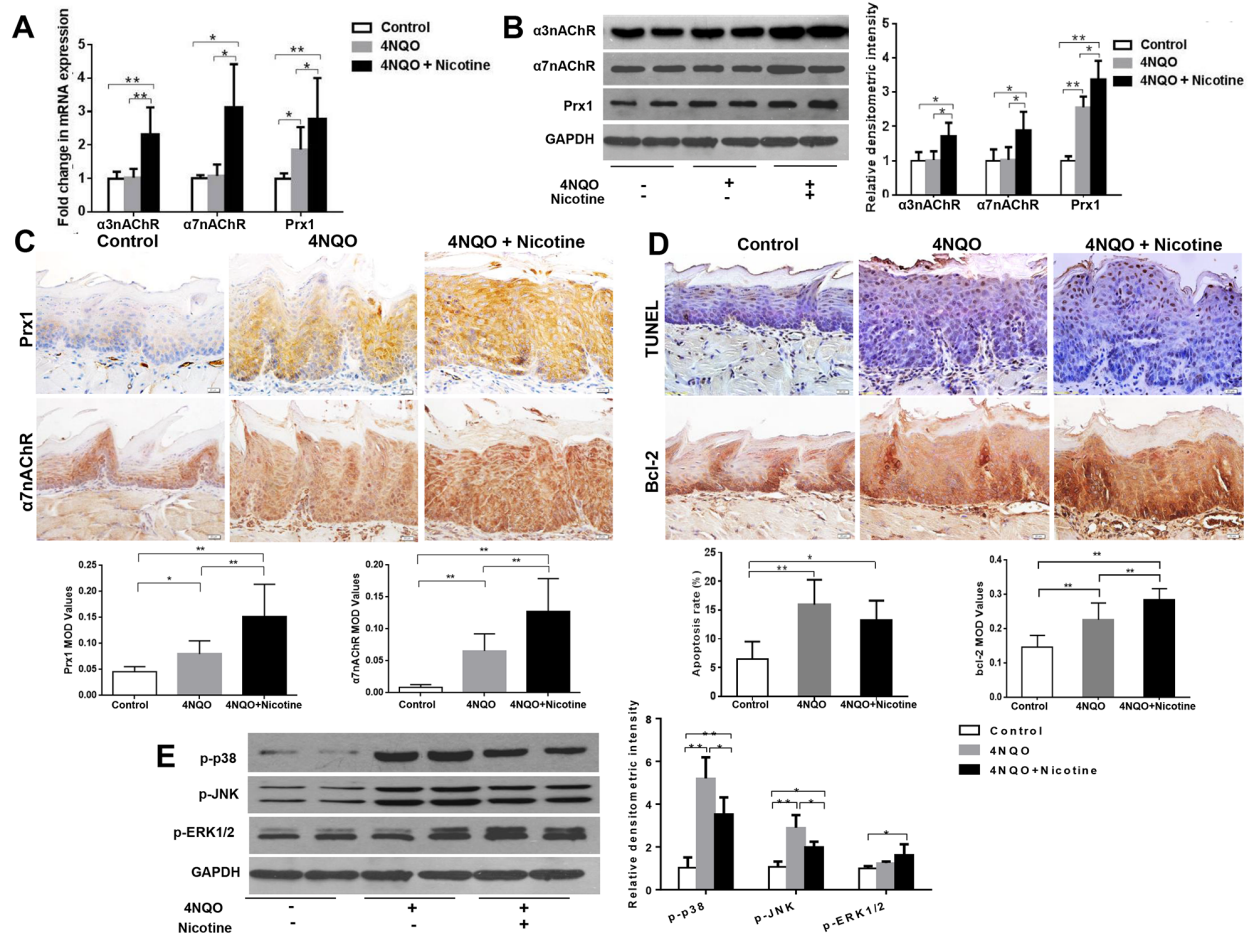
Effects of 4NQO and 4NQO + nicotine on Prx1, α3nAChR, α7nAChR, apoptosis and MAPK in Prx1 wild-type (Prx1^+/+^) mice **(A)** mRNA expression of α3nAChR, α7nAChR and Prx1; **(B)** protein expression of α3nAChR, α7nAChR and Prx1; **(C)** expression of Prx1 and α7nAChR detected by IHC; **(D)** apoptosis detected by TUNEL and Bcl-2 expression; **(E)** phosphorylation of p38, JNK and ERK1/2. The values are expressed as mean; *bars*, ± SE. * *P* < 0.05; ** *P* < 0.01.

In wild-type mice, the expression levels of Prx1, α3nAChR and α7nAChR were significantly increased in mice treated with 4NQO + nicotine when compared to those treated with 4NQO only (Figure 5A, 5B and 5C). As shown in Figure 5D, 4NQO and 4NQO + nicotine activated apoptosis when compared to control animals. The animals treated with 4NQO + nicotine had lower apoptosis rates when compared to those treated with 4NQO only (*P* > 0.05; Figure 5D). Moreover, the expression of Bcl-2 increased in animals treated with 4NQO + nicotine when compared to those treated with 4NQO only (Figure 5D). In addition, 4NQO + nicotine decreased expression of p-p38 and p-JNK (*P* < 0.05) and increased expression of p-ERK1/2 (*P* = 0.77) when compared to animals treated with 4NQO alone (Figure 5E).

### Prx1 knockdown increases cell apoptosis in mouse tongue precancerous lesions

**Figure 6:**
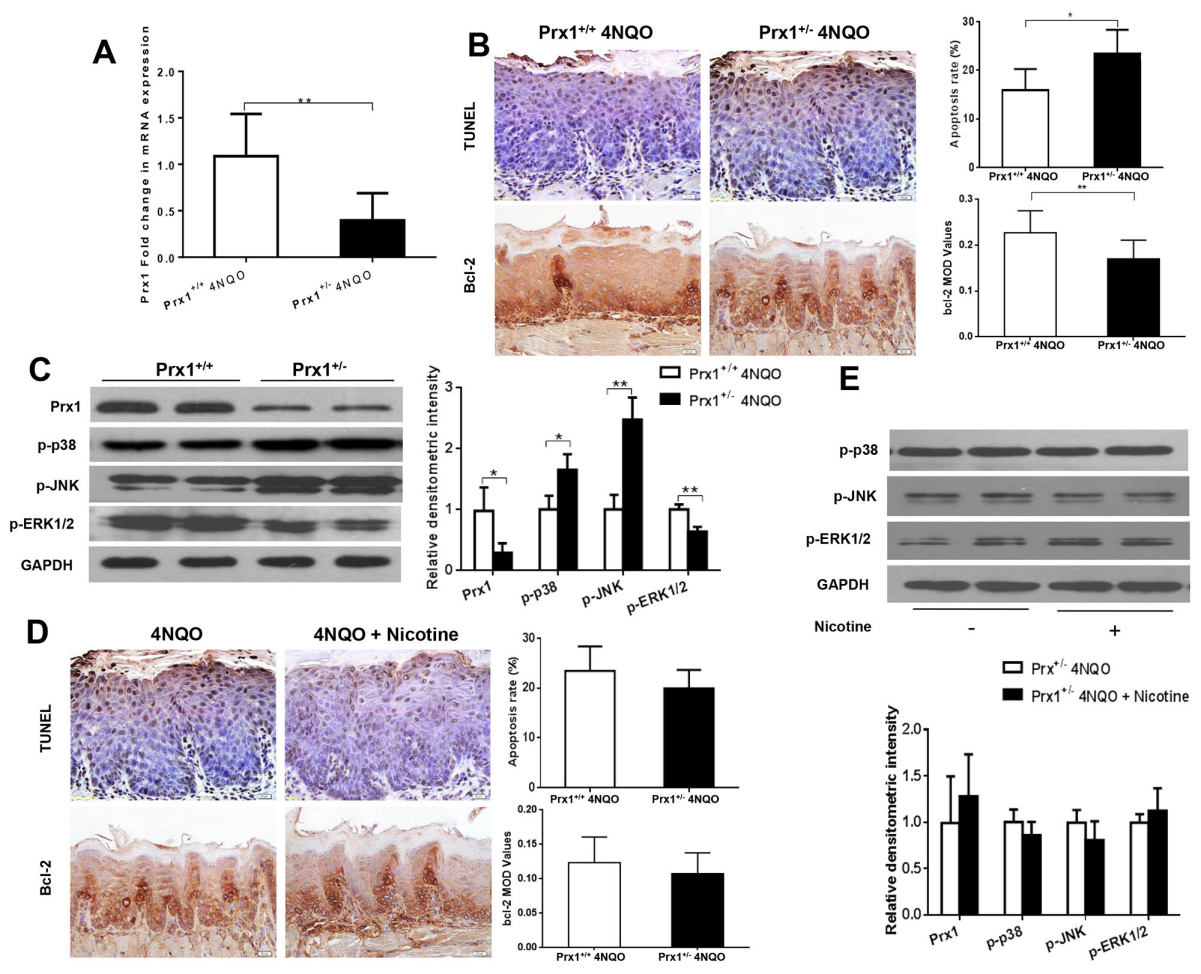
Effects of 4NQO and 4NQO + nicotine on Prx1, apoptosis and MAPK in Prx1 knockdown (Prx1^+/-^) mice **(A)** mRNAexpression of Prx1; **(B)** apoptosis detected by TUNEL and Bcl-2 expression in animals treated with 4NQO; **(C)** protein expression of Prx1, p-p38, p-JNK and p-ERK1/2 in animals treated with 4NQO; **(D)** apoptosis detected by TUNEL and Bcl-2 expression in Prx1 knockdown mice treated with 4NQO or 4NQO plus nicotine; and **(E)** protein expression of Prx1, p-p38, p-JNK and p-ERK1/2 in Prx1 knockdown mice treated with 4NQO or 4NQO plus nicotine. The values are expressed as mean; *bars*, ± SE. * *P* < 0.05; ** *P* < 0.01.

In Prx1 knockdown mice, the expression of Prx1 was significantly reduced compared to wild-type mice (*P* < 0.05) (Figure 6A and 6C). The apoptosis rate was increased and the expression of Bcl-2 was decreased by 4NQO in Prx1 knockdown mice when compared to wild-type mice (*P* < 0.05) (Figure 6B). The phosphorylation of p38 and JNK was increased and the phosphorylation of ERK1/2 was decreased by 4NQO in Prx1 knockdown mice when compared to wild-type mice (Figure 6C). In Prx1 knockdown mice, no significant difference in apoptosis and the expression of Bcl-2 was observed between 4NQO only and 4NQO + nicotine treatments (Figure 6D). In addition, there was no significant difference in phosphorylation of p38 JNK and ERK1/2 between the two treatment groups (Figure 6E).

## DISCUSSION

Prx1 plays an oncogenic role in nicotine-related oral carcinogenesis. In this study, we found that nicotine inhibited apoptosis by upregulating Prx1 in oral precancerous lesion cells and 4NQO-induced precancerous lesions in mouse tongue. Nicotine can promote carcinogenesis through its genotoxic effects, facilitating cancer cell growth, apoptosis, metastasis, resistance to therapies, and creating an immune suppressed microenvironment [7]. In this study, we found that nicotine contributes to epithelial precancerous growth, stimulates susceptibility to 4NQO-induced tongue precancerous lesions and accelerates its progression in Prx1 wild-type mice. However, nicotine alone does not induce precancerous growth. These results demonstrate that nicotine serves as a tumor promoter in the development of oral precancerous lesions in mice exposed to 4NQO.

Cell apoptosis is a critical cellular event in oral carcinogenesis. Bcl-2 has been identified as a key regulator of apoptosis, which is overly expressed in OLK and oral lichen planus [20, 21]. Apoptosis-related upstream regulators, such as Aurora A, phospho-MEK1/2 and phospho-ERK1/2, are closely correlated with malignant transformation of OLK [22]. One of the tumor-promoting activities of nicotine is to inhibit apoptosis. Our study shows that nicotine significantly inhibits apoptosis in DOK cells and 4NQO-induced tongue precancerous lesions in Prx1 wild-type mice. Moreover, we found that after Prx1 knockdown, the inhibition of apoptosis by nicotine is attenuated. Prx1 knockdown inhibits precancerous lesion development. The cell apoptosis is increased by Prx1 knockdown both *in vivo* and *in vitro*. These results demonstrate that nicotine inhibits apoptosis by upregulating Prx1 in oral precancerous lesions.

Prx1 is a member of the thiol-specific peroxidases family and plays diverse roles including H_2_O_2_ scavenger, redox signal transducer, molecular chaperone and oncogene [23]. Prx1 regulates several cell biological processes including cell differentiation, proliferation, and apoptosis. Some studies show that Prx1 protects cancer cells by suppressing oxidative-stress associated apoptosis [24, 25]. Nicotine can induce oxidative stress and has a positive feedback on regulating Prx1 in OSCC and oral precancerous lesions [26, 27]. In this study, we found that nicotine upregulates α3nAChR and α7nAChR in oral precancerous lesion cells. To further elucidate the association between nAChR and Prx1, we assessed Prx1 and apoptosis after α3nAChR and α7nAChR were blocked respectively. α-Neurotoxin, such as α-BTX, is a specific inhibitor of α7nAChR and binds tightly to α7nAChR through interacting between Tyr^184^ and local residues to high-affinity subtype-selective α-BTX binding. In this study, we also found that α-BTX significantly reduces nicotine-induced overexpression of α7nAChR and Prx1, and activates apoptosis. α3nAChR knockdown, however, has little effects on Prx1 and apoptosis.

Nicotine promotes lung carcinogenesis through binding to α3, α5, β4 and α7nAChR and subsequently activating cell proliferation, apoptosis, angiogenesis and tumor invasion [28]. Activation of cell membrane nAChRs via α7 and β2 subunits is associated with increased expression of cyclin D1 and phosphorylation of ERK1/2, inhibition of mPTP opening and resistance to H_2_O_2_-induced apoptosis in oral and lung cancers [15]. Activation of α7, α3, α4 and α9 nAChR produces growth-promoting and anti-apoptotic signaling that implements the tumor-promoting action of nicotine on lung cells [16]. There are two different pathways involved in cell apoptosis: the extrinsic pathway and the intrinsic pathway [29]. The intrinsic apoptotic pathway is controlled by Bcl-2 protein family through mitochondrial outer membrane permeabilization. The Bcl-2 family is composed of about 24 members including cell death suppressors, such as Bcl-2 and cell death inducer, such as Bax [30]. Nicotine increases Bcl-2 expression in breast cancer cell line MCF-7 cells [31]. Cytocrome c is the key component binding to form apoptosome in cytosolic and initiates caspase activation [30]. α7nAChR is expressed on mitochondrial outer membrane. The activation of α7nAChR on mitochondrial can prevent cytochrome c release, thus, blocks intrinsic apoptosis [32, 33]. In addition, activation of nAChRs can activate p38 MAPK, AKT, RAS/RAF/MERK/ERK/JAK2 signaling pathways. Theses molecular events are involved in nicotine-promoted carcinogenesis [34-37].

ASK1 is a member of mitogen-activated protein kinase kinase kinase family (MAP3K), also called MAP3K5. ASK1 activates downstream MAPK in response to oxidative stress and induces the cascades of cellular responses including apoptosis, differentiation, cell survival, and production of inflammatory cytokines [38-40]. Numerous studies show that the ASK1/MAPK pathway is involved in tumorigenesis through regulating inflammation, cell proliferation and apoptosis [41, 28]. In our previous study, we found that Prx1 inhibited ASK1-induced apoptosis in OLK [19]. However, the association between Prx1 and ASK1’s downstream MAPK has not been fully studied. The current study extends the previous pilot investigation through a more rigorous mechanistic study. Our data show that nicotine inhibits apoptosis through upregulation of α7nAChR and Prx1, and suppression of p38 MAPK, JNK and ERK signaling.

In conclusion, this is the first study to elucidate the function of nAChR/Prx1 axis in nicotine-related oral precancerous lesions in mice. Our results indicate that nicotine promotes oral precancerous growth through suppression of apoptosis via upregulating α7nAChR and Prx1. These findings are important because they provide experimental supports that the nAChR/ Prx1 axis may serve as a new target for treatment of oral OLK and thus, prevent OSCC in humans.

## MATERIALS AND METHODS

### Chemicals and reagents

4NQO, nicotine and α-Bungarotoxin (α-BTX) were obtained from Sigma-Aldrich (St. Louis, MO, USA). TRIzol and Lipofectamine™ 2000 were obtained from Invitrogen Life Technologies (Grand Island, NY, USA). cDNA Reverse Transcription Kit and SYBR Green Dye reagent were obtained from Applied Biosystems (Grand Island, NY, USA). Antibodies against Prx1, α3nAChR, α7nAChR and Bcl-2 were purchased from Abcam (Cambrige, MA, USA); antibodies against p-p38, p-JUN N-terminal kinase (JNK), p-extracellular signal-regulated kinase (ERK) and all secondary antibodies were purchased from Cell signaling Technology (Beverly, MA, USA); antibodies against GAPDH were purchased from Sigma-Aldrich (St. Louis, MO, USA). Enhanced chemiluminescence reagent was purchased from Amersham Biosciences (Pittsburgh, PA, USA). Prx1 shRNA lentivirus plasmid and control shRNA lentivirus plasmid-A were purchased from Suzhou gemma gene (Suzhou, China). α3nAChR shRNA plasmid and control plasmid were purchased from Santa Cruz Biotechnology (Dallas, Texas, USA).

### Cell culture and treatment

The human dysplastic oral keratinocyte (DOK) cell line was provided by Dr. Xiaoxin Chen in North Carolina Central University, USA. DOK cells were maintained in DMEM-High glucose supplemented with 10% (v/v) fetal bovine serum (FBS) (Gibco, USA) containing 100 units/mL penicillin, and 100 μg/mL streptomycin, in a 5% CO_2_ atmosphere at 37 °C. For nicotine treatment, cells received 1 μmol/ml nicotine for 7 days. To inhibit α7nAChR expression, cells were treated with 1 umol/ml α-BTX (a specific inhibitor of α7-nAChR) plus nicotine. To establish Prx1 or α3nAChR knockdown cell lines, Prx1 shRNA lentivirus plasmid, control shRNA lentivirus plasmid-A, α3nAChR shRNA plasmid, and control shRNA plasmid-A were respectively transfected to cells using Lipofectamine™ 2000 according to the manufacturer’s instructions. After transfection for 48 h, stable transfected cell lines were selected by puromycin (1ug/ml) for 10 days. The efficiency of Prx1 shRNA knockdown was determined by RT-PCR and Western Blot analyses. To detect apoptosis, cells were seeded on the glass slide fixed by paraformaldehyde. Apoptosis was examined by using In Situ Cell Death Detection Kit, POD (Roche, Germany) according to the manufacturer’s instruction. Finally, the apoptosis rates were detected by Image Pro.

### Animal bioassay

Wild type C57BL/6 mice were purchased from Vital River Laboratory Animal Technology (Beijing, China). Prx1 knockout mice, 6-8 weeks old, were used in this study [42]. All animals were kept in accordance with institutional guidelines under standard conditions. The experimental protocol was approved by the ethical committee for animal use. The experimental mice were randomly divided into eight groups (Table 1). Wild type mice (Prx1^+/+^) in Groups 1, 3, 5 and 7 received the treatment of the vehicle (distilled water; Group 1), 5% nicotine smeared on tongue mucosa (x3/week; Group 3), 50 μg/mL 4NQO (Group 5), or 4NQO + nicotine (Group 7). The Prx1 knockout mice (Prx1^+/-^) were randomized into four groups (Groups 2, 4, 6 and 8). At the end of bioassay (16 weeks), mouse tongues were removed after euthanasia and cut in half. One half was immediately stored in liquid nitrogen for future molecular/cellular analysis, and the other half was fixed in formalin to prepare paraffin-embedded tissue blocks.

### TUNEL staining

Apoptosis was examined by using In Situ Cell Death Detection Kit, POD (Roche, Germany) according to the manufacturer’s instruction. The slides were deparaffinized with histoclear and rehydrated in graded ethanol (100–70%). In the cell experiments, cell growing on the glass slide was fixed by paraformaldehyde. The specimens were subjected to PBS washing for 2 times with 5 min each time, and incubation at 37 °C for 15 min by proteinase K. Dry specimens were dropped in 50 μL of TUNEL reaction mixture, hydrated at light-free condition and incubated at 37 °C for 60 min. The PBS washed for 3 times with 5 min each time. The dry specimens were then dropped in 50 μL of converter-POD and incubated at 37 °C for 60 min. Freshly prepared DAB solution was used to incubate the specimens for 20 minutes. The apoptosis rates were detected by Image Pro.

### Quantitative real-time PCR

Total RNA was extracted from the mouse tongue tissues or DOK cells using TRIzol according to the manufacturer’s instructions. cDNA was synthesized with the High-Capacity cDNA Reverse Transcription Kit. SYBR Green Dye reagent was used to quantify the products formed during the Real-Time PCR reaction. For data analysis, the 2^-ΔΔCt^ method was used with normalization of raw data to the housekeeping gene GAPDH. The experiments were repeated at least three times. The sequence of primers was: GAPDH-F, 5’-aggtcggtgtgaacggatttg-3’,GAPDH-R, 5’-tgtagaccatgtagttgaggtca-3’; α3nAChR-F, 5’-ggacgggatgtgtggttact-3’; α3nAChR-R, 5’-tggcttctttgatttctggtg-3’; α7nAChR-F, 5’-aaactcacagatgggcaagg-3’; α7nAChR-R, 5’-ccgtaagcaacacgactgac-3’; Prx1-F, 5’-gggtattcttcggcagatca-3’, Prx1-R, 5’-tccccatgtttgtcagtgaa-3’. In *in-vivo* experiments, the sequence of primers was: GAPDH-F, 5’-aggtcggtgtgaacggatttg-3’, GAPDH-R, 5’-tgtagaccatgagttgaggtca-3’; α3-nAChR-F, 5’-atggaaaccaacctgtggct-3’, α3-nAChR-R, 5’-aaatccccatcggcgttgtt-3’; α7-nAChR-F, 5’-gcaacatctgattccgtgcc-3’; α7-nAChR-R, 5’-tgatcctggtccacttaggc-3’; Prx1-F, 5’-aatgcaaaaattgggtatcctgc-3’, Prx1-R, 5’-cgtgggacacacaaaagtaaagt-3’.

### Western blot analysis

Proteins were extracted from mouse tongue tissues or DOK cells with immunoprecipitation assay buffer. Protein concentration was determined using the Lowry method. Equal amounts of protein were separated on 12% SDS-PAGE gels and blotted onto nitrocellulose membranes. After incubated with primary antibody and horseradish peroxidase-conjugated secondary antibodies, immunoreactive bands were detected with enhanced chemiluminescence reagent. The following antibodies were used: Prx1 (Abcam, USA, 1:1000), α3nAChR (Abcam, USA, 1:500), α7nAChR (Abcam, USA, 1:1000), p38, p-p38, JNK, p-JNK (CST, USA, 1:1000), ERK1/2, p-ERK1/2 (CST, USA, 1:2000) and GAPDH (Sigma, USA, 1:2000). The experiments were repeated at least three times.

### Immunohistochemistry

Mouse tongue tissues were fixed in 10 % neutral formalin for 24 h, serially sectioned at 4 μm, and processed for immunohistochemistry. Deparaffinised sections were briefly heated for 10 min in a pressure cooker containing 10 mM citrate buffer (pH 6.0) for antigen retrieval, and followed by 3 % H_2_O_2_ in 0.1 M TBS (pH 7.4) for 15 min to quench endogenous peroxidases. The sections were then treated with protein block solution (Boshide, China) for 20 min and incubated with anti-Bcl-2 antibody (1:200), Prx 1 (1:50) and α7nAChR (1:50) overnight at 4°C. Incubation with primary antibody was followed by incubation with secondary anti-rabbit GT VisionTM polymer (Gene Tech, South San Francisco, California, USA) for 30 min. Finally, slides were treated with chromogen diaminobenzidine (DAB) (Dako, Carpinteria, CA, USA), counterstained with haematoxylin, dehydrated, and mounted for further Olympus BX61 microscope observation (Olympus, Tokyo, Japan). To assess the protein expression in each tongue, five random areas within a section were selected at 200 x× magnification. The arithmetic mean proportion of positive cells of the five areas represented the protein expression.

### Statistical analysis

Group differences were analyzed for statistical significance by Chi-Square test, ANOVA and t-test. Differences were considered statistically significant at *P* < 0.05. All *P* values were two-sided. The analyses were conducted using SPSS 17.0.

## SUPPLEMENTARY MATERIALS FIGURE



## Abbreviations

nAChRs: nicotinic acetylcholine receptors
Prx1: peroxiredoxin 1
OSCC: oral squamous cell carcinoma
4NQO: 4-nitroquinoline 1-oxide
JNK: JUN N-terminal kinase
ERK: extracellular signal-regulated kinase
MAPK: mitogen-activated protein kinases
OLK: oral leukoplakia
EGF: epidermal growth factor
PI3K: phosphatidylinositol-4,5-bisphosphate 3-kinase
ASK1: apoptosis signal-regulating kinase 1
GAPDH: glyceraldehyde-3-phosphate dehydrogenase
DOK: dysplastic oral keratinocyte
mPTP: mitochondrial permeability transition pore
α-BTX: α-Bungarotoxin
Bcl-2: B-cell lymphoma 2
MEK: MAPK/ERK kinase
PLC: phospholipase C
PKC: protein kinase C
JAK2: Janus kinase 2
