# Supplementary material for: Nicotine suppresses apoptosis by regulating α7nAChR/Prx1 axis in oral precancerous lesions
Source: Oncotarget. 2017 Aug 24;8(43):75065–75. doi: 10.18632/oncotarget.20506 (PMC5650400; doi:10.18632/oncotarget.20506)
Supplement: Supplementary file 1 [file oncotarget-08-75065-s001.pdf]

Nicotine suppresses apoptosis by regulating α7nAChR/Prx1 axis in oral precancerous lesions

SUPPLEMENTARY MATERIALS

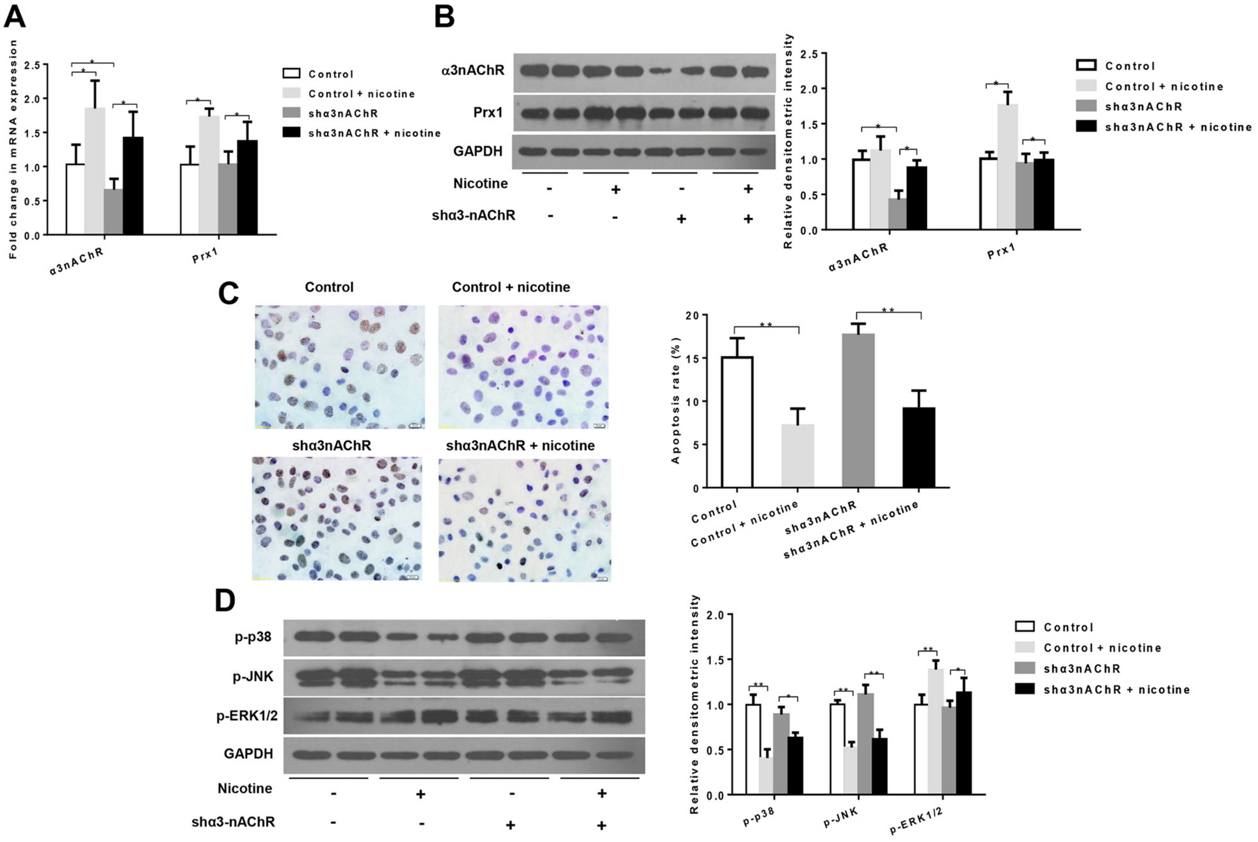

Supplementary Figure 1: Effects of α3nAChR knockdown on Prx1 and opoptosis in nicotine-treated DOK cells. (A) mRNA expression of α3nAChR and Prx1; **(B)** protine expression of α3nAChR and Prx1; **(C)** apoptosis rate detected by TUNEL; and **(D)** phosphorylation of p38, JNK and ERK1/2. The values are expressed as mean; bars, ± SE. $*P < 0.05$; $**P < 0.01$.
